# Knowledge, attitude and practice towards COVID-19 among individuals with associated comorbidities

**DOI:** 10.25122/jml-2020-0184

**Published:** 2021

**Authors:** Shazina Saeed, Aanchal Anant Awasthi, Dhruv Nandi, Karunanidhi Kaur, Shamimul Hasan, Rajiv Janardhanan

**Affiliations:** 1. Laboratory of Disease Dynamics and Molecular Epidemiology, Amity Institute of Public Health, Amity University, Uttar Pradesh, Noida, India; 2. Health Data Analytics and Visualization Environment Laboratory, Amity Institute of Public Health, Amity University, Uttar Pradesh, Noida, India; 3. Department of Oral Medicine and Radiology, Faculty of Dentistry, Jamia Millia Islamia, New Delhi, India

**Keywords:** comorbidities, COVID-19, India, public health

## Abstract

The recent outbreak of coronavirus disease 2019 (COVID-19) is the worst global crisis after World War II. Since the vaccine trials are still ongoing, the national lockdowns have been the most effective way to contain its spread. The pandemic has been extremely stressful and full of anxiety for those with comorbidities as they are the most vulnerable to the COVID-19 infections. Various efforts to assess and enhance the knowledge, attitudes, and practice, especially the high-risk groups, are critical to managing the COVID-19 pandemic successfully. A cross-sectional online survey was carried out using a self-designed questionnaire. A total of 383 participants who were 30 years or older, with at least one comorbidity, were included in the study. The mean age of the participants was 50.63±11.83 years. The most common comorbidities among the included participants were hypertension, followed by diabetes mellitus and thyroid disorders (48.5%, 44.7% and 23.3%, respectively). Our study also showed the education (p=0.004) and occupation (p=0.04) had a significant association with the attitude and practices towards the COVID-19 pandemic. In conclusion, our study showed that a high level of knowledge, attitude, and practices are the backbone to combat a global crisis like COVID-19.

## Introduction

On 31^st^ December 2019, the Wuhan health commission in the Hubei province of The Republic of China alerted the National Health Commission, Chinese Centre for Disease Control and Prevention (CDC), and the World Health Organization (WHO) of an array of 27 patients with pneumonia of unidentified origin [1]. The first reported case was on the 8^th^ December 2019, with most patients either working or living in the neighborhood of Huanan Seafood Market. A peculiar coronavirus (initially termed as 2019-nCoV by WHO) was detected from the patient’s throat swab samples on 7^th^ January. The Coronavirus Study Group termed it as severe acute respiratory syndrome coronavirus 2 (SARS-CoV-2), and the WHO framed the term coronavirus disease 2019 (COVID-19) [2].

SARS-CoV-2 is a new public health crisis threatening the world with the emergence and spread of the COVID-19 infection. WHO affirmed COVID-19 as a pandemic on 11^th^ March 2020 [3]. As of 2^nd^ December 2020, SARS-CoV-2 is reported to have a widespread incidence in 215 countries globally, with a total of 18,338,454 infected cases, 44,754,905 recovered cases, and 1,494,868 deaths [4]. COVID-19 presents with asymptomatic, mild, or severe pneumonia-like symptoms. COVID-19 patients with comorbidities like diabetes, hypertension, renal, respiratory, and cardiovascular disorders, malignancies, HIV, and others are classified as high-risk individuals. They are most susceptible to develop septic shock, acute respiratory distress syndrome, electrolyte imbalances (metabolic acidosis), and coagulation disorders, eventually resulting in the patient’s death [5].

It is well studied now that the SARS-CoV-2 virus utilizes the Angiotensin-converting enzyme 2 (ACE2) receptors found at the surface of the host cells to gain entry inside the cell. Certain comorbidities like hypertension and diabetes are associated with a strong ACE-2 receptor expression and release of increased proprotein convertase levels that encourage the viral entry into the host cells. COVID-19 patients with comorbidities lead a life with a vicious infectious circle and are at increased risk for significant morbidity and mortality rates [5].

Knowing that India is a hotspot of comorbid disorders, including cardiovascular disorders (CVDs), hypertension and diabetes, especially in older people [6]. The case fatality rate or mortality of COVID-19 is linked with the vulnerability and vulnerable groups [7]. The key variables conferring vulnerabilities appear to be age, hypertension, diabetes, chronic heart/lung/renal/hepatic disease states, and many more. Person-to-person transmission (community spread) is currently ongoing in India, making it necessary to control the disease to avoid its rapid spread throughout the country. An effective disease management warrants community compliance for preventive and control actions. This compliance is highly contingent on the population’s knowledge, attitudes, and practices (KAP) towards COVID-19. In 2014, Blendon RJ *et al*. have already shown that the knowledge level and attitudes towards infectious diseases are associated with the level of panic among the population, which can further confound efforts to avert the dissemination of the disease, encouraging alternate management protocols [8].

Hence, it is imperative for the comorbid individual to adopt vigilant preventive measures and undergo scrupulous management. Keeping this in mind, we conducted this study to evaluate the knowledge, attitude, and practice in patients with comorbidities towards the COVID-19 pandemic.

## Material and Methods

This was a cross-sectional online survey carried out among 260 Indian residents. A self-designed structured questionnaire was developed for this study. This structured questionnaire was then converted into Google forms for online data collection. Participants aged 30 years and above and having any comorbid condition were included in the study. At first, the questionnaire was forwarded to authors’ known contacts using an email list and Whatsapp contacts with the request to forward it to their known contacts and so on. Informed consent was obtained from each participant before data collection.

The online self-reported questionnaire consisted of five sections to collect demographic details of the participants along with knowledge, attitude about COVID-19, and safe practice measurements during the outbreak. Information about comorbid conditions, duration of comorbidities, and challenges faced by them to manage their comorbid condition during this unpredictable time were also obtained from participants. To get the overall knowledge, attitude, and practice status of participants, each correct/positive response was assigned a value of 1; otherwise, the assigned value was 0. Further, these scores were added to the obtained total KAP scores for each section separately. Total KAP scores were divided into two categories as adequate/positive based on their respective second quartile. Exploratory data analysis was performed to explore the distribution of characteristics under study, and the Chi-square test of association was used to compute the association between categorical variables. Multi-variable binary logistic regression was used to identify factors associated with adequate knowledge. A p-value of less than 0.05 was considered statistically significant. Data were retrieved into Microsoft Excel and analyzed using the IBM SPSS software (IBM SPSS Statistics for Windows, Version 23.0. Armonk, NY: IBM Corp).

## Results

### Demographic characteristics of the participants

**Figure 1.**
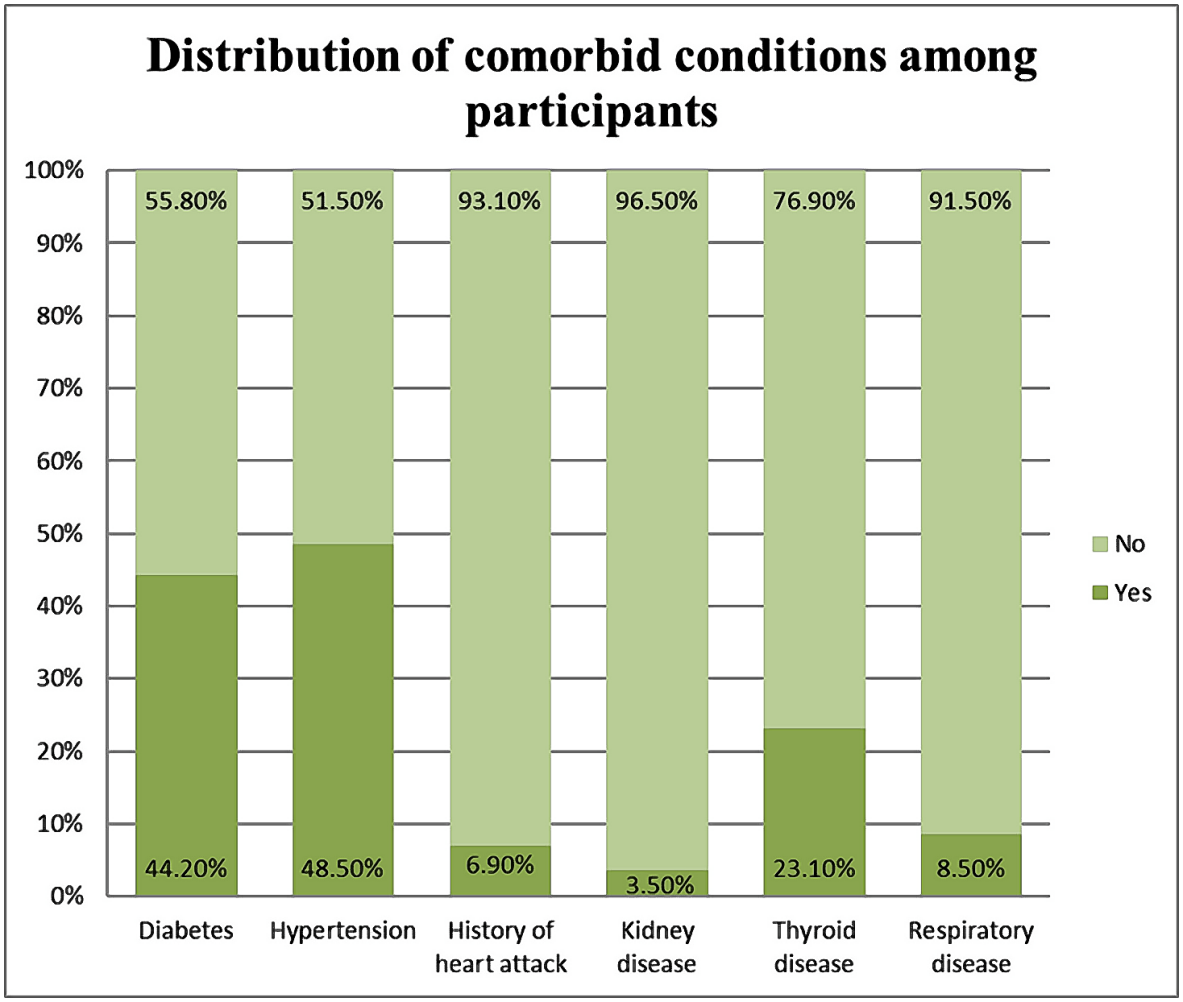
Distribution of comorbid conditions among participants (n=260).

A total of 300 participants completed the questionnaire. After excluding 40 respondents who reported to be below 30 years old, the final sample consisted of 260 participants. The majority of the participants were 50 years or older (50.8%), predominantly females (58.5%). Most of the participants were post-graduates or had their doctorate degree completed (43.5%). 91.2% of participants were married, and 41.9% were working, most of them acquiring a monthly salary of 31,000 Indian rupees (INR) and above (80.9%). The participants were predominantly suffering from one comorbid condition (71.5%) and were diagnosed less than 10 years ago (73.8%). The most common comorbidities among the included participants were hypertension, followed by diabetes mellitus and thyroid disorders (48.5%, 44.7%, and 23.3%, respectively), as shown in Figure 1.

### Knowledge, attitude and practice towards COVID-19

**Figure 2.**
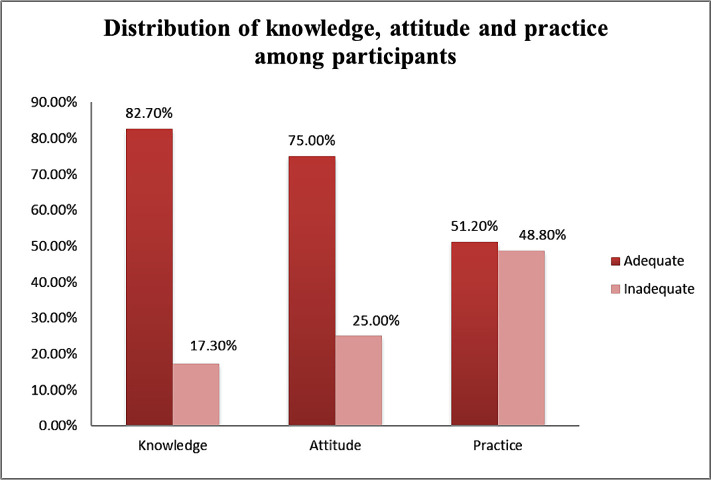
Distribution of knowledge, attitude, and practice scores among participants.

The adequate overall knowledge, attitude, and practice scores of the participants in terms of COVID-19 were 82.7%, 75.0% and 51.2%, respectively, as shown in Table 1 and Figure 2. The knowledge, attitude and practice towards COVID-19 was seen to be adequate among participants aged 50 years or older as compared to less than 50 years old participants (89.4% vs. 75.8%), 78.8% vs. 71.1%, 51.5% vs. 50.8%, respectively) (Table 2). Gender-wise, no difference was found concerning knowledge, attitude, and practice about COVID-19. Similarly, we did not find a significant association between KAP and educational status (p=0.052, 0.507 and 0.280, respectively for KAP). Participants suffering from more than one comorbid condition (93.2%) reported adequate knowledge as compared to those having one comorbid condition (78.5%). There was no significant association found based on the duration of diagnosis of the comorbid condition. Among all the participants, diabetic and hypertensive individuals showed better knowledge towards COVID-19 (89.6% and 88.9%, respectively). The age of the participants (p=0.011), along with the diagnosis of their comorbid conditions (p=0.005), was found to be significantly associated with the knowledge score. Also, individual comorbid conditions like diabetes (p=0.009), hypertension (p=0.010), and thyroid disease (p=0.003) were significant regarding knowledge towards COVID-19 (Table 2).

**Table 1.** Frequencies and percentages of knowledge, attitude, and practice about COVID-19 among the participants.

Questions		^Preferred Response (%)^	^Not preferred Response (%)^
Knowledge-based questions			
**1.**	**Do you know that your condition is at a high risk for SARS-Cov-2 infection?**	214 (82.3)	46 (17.7)
**2.**	**If you and your family are experiencing COVID-19 symptoms, how long should the quarantine period be?**	151 (58.1)	109 (41.9)
**3.**	**Which are the most common symptoms that you need to be aware of as a potential SARS-Cov-2 infection?**	239 (91.9)	21 (8.1)
**4.**	**Do you think that you are preventing COVID-19 transmission by avoiding hand-face-mouth contact?**	237 (91.2)	23 (8.8)
**Atttitude-based questions**			
**5.**	**Do you think the national lockdown due to the COVID-19 pandemic is affecting you and has worsened your condition?**	Positive attitude (%)	Negative attitude (%)
		131 (50.4)	129 (49.6)
**6.**	**Do you think keeping a positive attitude is the only way to combat the current situation?**	205 (78.8)	55 (21.2)
**7.**	**Which activities do you think are important in preventing COVID-19 transmission?**		
	a) Remove shoes/slippers upon entering homes	225 (86.5)	35 (13.5)
	b) Clean fruits and vegetables aggressively	226 (86.9)	34 (13.1)
	c) Keeping groceries in sunlight for at least 4 hours	46 (17.7)	214 (82.3)
**8.**	**You are aware that the pandemic is still spreading; however, if the lockdown is lifted, will you still maintain social distancing?**	Yes, I will still maintain social distancing, take all precautions and stay positive.	No, I am tired of this lifestyle, I want things to be back to normal. I feel depressed and lonely and I will visit my friends and family.
		217 (83.5)	43 (16.5)
**Practice-based questions**			
**9.**	**Are you able to monitor your health during this lockdown due to the COVID-19 pandemic?**	^Positive practice (%)^	^Negative practice (%)^
		189 (72.7)	71 (27.3)
**10.**	^Are you facing difficulties with the following during the COVID-19 lockdown?^		
	Access to medicines	169 (65.0)	91 (35.0)
	Access to lab investigations	125 (48.1)	135 (51.9)
	Access to doctors	103 (39.6)	157 (60.4)
**11.**	**How are you protecting yourself from the COVID-19 infection?**		
	Social distancing	259 (99.6)	1 (0.4)
	General hygiene	245 (94.2)	15 (5.8)
	Face mask	254 (97.7)	6 (2.3)
	Washing hands frequently for 30–40 seconds	224 (86.2)	36 (13.8)
	Immune-boosting diet	222 (85.4)	38 (14.6)
	Regular exercises/yoga/meditation	152 (58.5)	108 (41.5)

**Table 2.** The association between managing comorbidities during the COVID-19 pandemic and related socio-demographic variables.

S.no.	Socio-demographic variable	Total	Monitoring health		P-value	Access to lab investigation		P-value	Access to medicines		P-value	Access to doctors		P-value
		N (%)	Yes N (%)	No N (%)		Yes N (%)	No N (%)		Yes N (%)	No N (%)		Yes N (%)	No N (%)	
**1.**	**Age**													
	Less than or equal to 50 years	128 (49.2)	93 (72.7)	35 (27.8)	0.990	63 (49.2)	65 (50.8)	0.390	43 (33.6)	85 (66.4)	0.640	76 (59.4)	52 (40.6)	0.743
	More than 50 years	132 (50.8)	96 (72.7)	36 (27.3)		72 (54.5)	60 (45.5)		48 (36.4)	84 (63.6)		81 (57.6)	51 (42.4)	
**2.**	**Education**													
	Primary education	42 (16.2)	34 (81.0)	8 (19.0)	0.328	27 (64.3)	15 (35.7)	0.208	18 (42.9)	24 (57.1)	0.042	26 (62.0)	16 (38.0)	0.706
	Graduate	105 (40.4)	77 (73.3)	28 (26.7)		51 (48.6)	54 (51.4)		43 (41.0)	62 (59.0)		66 (62.9)	39 (37.1)	
	Postgraduate/Doctorate	113 (43.5)	78 (69.0)	35 (31.0)		57 (50.4)	56 (49.6)		30 (26.5)	83 (73.5)		65 (57.5)	48 (42.5)	
**3.**	**Occupation**													
	Working	109 (41.9)	75 (68.8)	34 (31.2)	0.232	59 (54.1)	50 (45.9)	0.545	42 (38.5)	67 (61.5)	0.310	71 (65.1)	38 (34.9)	0.183
	Non-working	151 (68.1)	114 (75.5)	37 (24.5)		76 (50.3)	75 (49.7)		49 (32.5)	102 (67.5)		86 (57.0)	65 (43.0)	
**4.**	**Average Monthly Income (INR)**													
	0–30,000	35 (19.1)	25 (71.4)	10 (28.6)	0.664	17 (48.6)	18 (51.4)	0.511	16 (45.7)	19 (54.3)	0.244	18 (51.4)	17 (48.6)	0.244
	31,000 and above	148 (80.9)	111 (75.0)	37 (25.0)		81 (54.7)	67 (45.8)		52 (35.1)	96 (64.9)		92 (62.2)	56 (37.8)	
**5.**	**Diagnosed with comorbidities**													
	One comorbid condition	186 (71.5)	130 (69.9)	56 (30.1)	0.108	99 (53.2)	87 (46.8)	0.505	61 (32.8)	125 (67.2)	0.237	110 (59.1)	76 (40.9)	0.515
	Multiple comorbid conditions	74 (28.5)	59 (79.7)	15 (20.3)		36 (48.6)	38 (51.4)		30 (40.5)	44 (59.5)		47 (63.5)	27 (36.5)	
**6.**	**Duration of diagnosis**													
	Less than or equal to 10 years	192 (73.8)	143 (74.5)	49 (25.5)	0.277	94 (49.0)	98 (51.0)	0.108	65 (33.9)	127 (66.1)	0.515	116 (60.4)	76 (39.6)	0.986
	More than 10 years	68 (26.2)	46 (67.6)	22 (32.4)		41 (60.3)	27 (39.7)		26 (38.2)	42 (61.8)		41 (60.3)	27 (39.7)	
**7.**	**Comorbid conditions**													
**A.**	**Diabetes**													
	Yes	115 (44.2)	90 (78.3)	25 (21.7)	0.073	56 (48.7)	59 (51.3)	0.354	45 (39.1)	70 (60.9)	0.214	68 (59.1)	47 (40.9)	0.713
	No	145 (55.8)	99 (68.3)	46 (31.7)		79 (54.5)	66 (45.5)		46 (31.7)	99 (68.3)		89 (61.4)	56 38.6)	
**B.**	**Hypertension**													
	Yes	126 (48.5)	98 (77.8)	28 22.2)	0.074	61 (48.4)	65 (51.6)	0.272	48 (38.1)	78 (61.9)	0.310	79 (62.7)	47 (37.3)	0.459
	No	134 (51.5)	91 (67.9)	43 (32.1)		74 (55.2)	60 (44.8)		43 (32.0)	91 (68.0)		78 (58.2)	56 (41.8)	
**C.**	**History of heart atack**													
	Yes	18 (6.9)	11 (61.1)	7 (38.9)	0.385	10 (55.6)	8 (44.4)	0.749	6 (33.3)	12 (66.7)	0.878	12 (66.7)	6 (33.3)	0.572
	No	242 (93.1)	178 (73.6)	64 (26.4)		125 (51.7)	117 (48.3)		85 (35.1)	157 (64.9)		145 (60.0)	97 (40.0)	
**D.**	**Kidney diseases**													
	Yes	9 (3.5)	9 (100.0)	0 (0.0)	0.136	2 (22.2)	7 (77.8)	0.093	3 (33.3)	6 (66.7)	>0.999	3 (33.3)	6 (66.7)	0.180
	No	251 (96.5)	180 (71.7)	71 (28.3)		133 (53.0)	118 (47.0)		88 (35.0)	163 (65.0)		154 (61.4)	97 (38.6)	
**E.**	**Thyroid disease**													
	Yes	60 (23.1)	34 (56.7)	26 (43.3)	^0.001^	39 (65.0)	21 (35.0)	^0.021^	17 (28.3)	43 (71.7)	0.217	36 (60.0)	24 (40.0)	0.945
	No	200 (76.9)	155 (77.5)	45 (22.5)		96 (48.0)	104 (52.0)		74 (37.0)	126 (63.0)		121 (60.5)	79 (39.5)	
**F.**	**Respiratory disease**													
	Yes	22 (8.5)	21 (95.5)	1 (4.5)	^0.012^	11 (50.0)	11 (50.0)	0.850	7 (31.8)	15 (68.2)	0.744	13 (59.1)	9 (40.9)	0.897
	No	238 (91.5)	168 (70.6)	70 (29.4)		124 (52.1)	114 (47.9)		84 (35.3)	154 (64.7)		144 (60.5)	94 (39.5)	

### Management of comorbidities during COVID-19 pandemic

**Figure 3.**
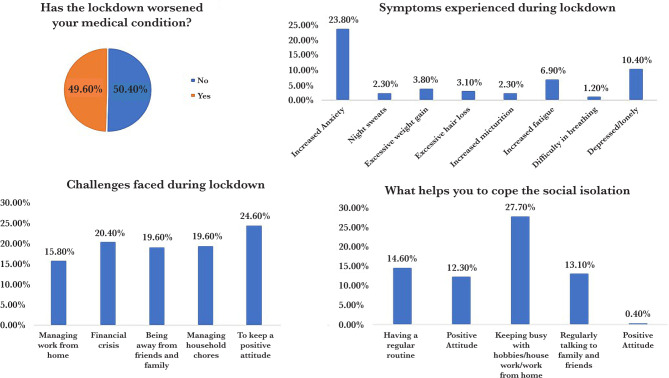
Challenges faced during the COVID-19 pandemic.

The management of comorbidities during COVID-19 is described as shown in Tables 2, 3 and Figure 3. Participants aged 50 years or older reported monitoring their health (36.9%) along with difficulty in accessing medicines (18.5%). Highly educated participants (31.9%) with a monthly income of 31,000/- and above (52.5%), suffering from multiple comorbid conditions (16.9%) with a duration of less than or equal to 10 years (48.8%) had difficulty in accessing medicines.

**Table 3.** Distribution and association of the KAP score of participants in relation to socio-demographic variables.

S.no.	Socio-demographic variable	Total	Knowledge score		P-value	Attitude score		P-value	Practice score		P-value
		N (%)	Adequate N (%)	Inadequate N (%)		Adequate N (%)	Inadequate N (%)		Adequate N (%)	Inadequate N (%)	
**1.**	**Age**										
	Less than or equal to 50 years	128 (49.2)	97 (75.8)	31 (24.2)	0.011	91 (71.1)	37 (28.9)	0.152	65 (50.8)	63 (49.2)	0.906
	More than 50 years	132 (50.8)	118 (89.4)	14 (10.6)		104 (78.8)	28 (21.2)		68 (51.5)	64 (49.5)	
**2.**	**Gender**										
	Male	108 (41.5)	90 (83.3)	18 (16.7)	0.818	82 (75.9)	26 (24.1)	0.771	57 (52.8)	51 (47.2)	0.659
	Female	152 (58.5)	125 (82.2)	27 (17.8)		113 (74.3)	39 (25.7)		76 (50.0)	76 (50.0)	
**3.**	**Education**										
	Primary education	42 (16.2)	39 (92.9)	3 (7.1)	0.052	29 (69.0)	13 (31.0)	0.507	17 (40.5)	25 (59.5)	0.280
	Graduate	105 (40.4)	89 (84.8)	16 (15.2)		82 (78.1)	23 (21.9)		54 (51.4)	51 (48.6)	
	Postgraduate/Doctorate	113 (43.5)	87 (77.0)	26 (23.0)		84 (74.3)	29 (25.7)		62 (54.9)	51 (45.1)	
**4.**	**Marital status**										
	Married	237 (91.2)	198 (83.5)	39 (16.5)	0.380	174 (73.4)	63 (26.6)	0.059	122 (51.5)	115 (48.5)	0.738
	Unmarried/others**	23 (8.8)	17 (73.9)	6 (26.1)		21 (91.3)	2 (8.7)		11 (47.8)	12 (52.2)	
**5.**	**Occupation**										
	Working	109 (41.9)	96 (88.1)	13 (11.9)	0.051	77 (70.6)	32 (29.4)	0.168	50 (45.9)	59 (54.1)	0.148
	Non-working	151 (68.1)	119 (78.8)	32 (21.2)		118 (78.1)	33 (21.9)		83 (55.0)	68 (45.0)	
**6.**	**Average Monthly Income (Rupees)**										
	0–30,000	35 (19.1)	31 (88.6)	4 (1.4)	0.378	24 (68.6)	11 (31.4)	0.340	20 (57.1)	15 (42.9)	0.329
	31,000 and above	148 (80.9)	122 (82.4)	26 (17.6)		113 (76.4)	35 (23.6)		71 (48.0)	77 (52.0)	
**7.**	**Diagnosed with comorbidities**										
	One comorbid condition	186 (71.5)	146 (78.5)	40 (21.5)	^0.005^	144 (77.4)	42 (22.6)	0.153	98 (52.7)	88 (47.3)	0.433
	Multiple comorbidities	74 (28.5)	69 93.2)	5 (6.8)		51 (68.9)	23 (31.1)		31 (42.0)	39 (58.0)	
**8.**	**Duration of diagnosis**										
	Less than or equal to 10 years	192 (73.8)	158 (82.3)	34 (17.7)	0.774	140 (72.9)	52 (27.1)	0.192	100 (52.1)	92 (47.9)	0.614
	More than 10 years	68 (26.2)	57 (83.8)	11 (16.2)		55 (80.9)	13 (19.1)		33 (48.5)	35 (51.5)	
**9.**	**Comorbid conditions**										
**A.**	**Diabetes**										
	Yes	115 (44.2)	103 (89.6)	12 (10.4)	^0.009^	83 (72.2)	32 (27.8)	0.349	54 (47.0)	61 (53.0)	0.228
	No	145 (55.8)	112 (77.2)	33 (22.8)		112 (77.2)	33 (22.8)		79 (54.5)	66 (45.5)	
**B.**	**Hypertension**										
	Yes	126 (48.5)	112 (88.9)	14 (11.1)	^0.010^	90 (71.4)	36 (28.6)	0.197	66 (52.4)	60 (47.6)	0.701
	No	134 (51.5)	103 (76.9)	31 (23.1)		105 (78.4)	29 (21.6)		67 (50.0)	67 (50.0)	
**C.**	**History of heart atack**										
	Yes	18 (6.9)	16 (88.9)	2 (11.1)	0.691	14 (77.8)	4 (22.2)	>0.999	10 (55.6)	8 (44.4)	0.699
	No	242 (93.1)	199 (82.2)	43 (17.8)		181 (74.8)	61 (25.2)		123 (50.8)	119 (49.2)	
**D.**	**Kidney diseases**										
	Yes	9 (3.5)	8 (88.9)	1 (11.1)	0.959	5 (55.6)	4 (44.4)	0.327	7 (77.8)	2 (22.2)	0.173
	No	251 (96.5)	207 (82.5)	44 (17.5)		190 (75.7)	61 (24.3)		126 (50.2)	125 (49.8)	
**E.**	**Thyroid disease**										
	Yes	60 (23.1)	42 (70.0)	18 (30.0)	^0.003^	49 (81.7)	11 (18.3)	0.174	30 (50.0)	30 (50.0)	0.838
	No	200 (76.9)	173 (86.5)	27 (13.5)		146 (73.0)	54 (27.0)		103 (51.5)	97 (48.5)	
**F.**	**Respiratory disease**										
	Yes	22 (8.5)	18 (81.8)	4 (18.2)	>0.999	16 (72.7)	6 (27.3)	0.797	13 (59.1)	9 (40.9)	0.436
	No	238 (91.5)	197 (82.8)	41 (17.2)		179 (75.2)	59 (24.8)		120 (50.4)	118 (49.6)	

** – Divorcee, Widowed, Separated.

Access to lab investigations and medicines were found to be difficult for diabetic (22.7%) and hypertensive (25.0) individuals. Accessibility to medicines was significantly associated with the education of the participants (0.042). Also, participants suffering from thyroid diseases (0.001) and respiratory diseases (0.012) were found to be significantly associated with monitoring health and accessibility to lab investigations.

### Independent predictors for adequate knowledge

During bivariate analysis, our study showed that age (p=0.005) and comorbidities like diabetes (p=0.011), hypertension (p=0.012) and thyroid perturbations (p=0.004) were significantly associated with responders’ knowledge. All these factors were analyzed in multivariable settings to identify the independent role of each of these factors after adjusting for these factors. We found that participants older than 50 years were 2.205 (1.072–4.534) times more knowledgeable about the COVID-19 pandemic compared to those participants aged less than or equal to 50 years. Similarly, participants suffering from diabetes and hypertension were found to be associated with more knowledge with an odds of 2.669 (1.187–6.002) and 2.412 (1.058–5.496), respectively, as shown in Table 4.

**Table 4.** Independent predictors for adequate knowledge.

Characteristic	Number (%)	Bivariate				Multi-variable			
		Odds Ratio	95% CI for Odds Ratio		P-value	Exp(B)	95% CI for Odds Ratio		P-value
			Lower	Upper			Lower	Upper	
**Age**									
Less than or equal to 50 years	128 (49.2)	1.000							
More than 50 years	132 (50.8)	2.694	1.357	5.348	.005	2.205	1.072	4.534	.032
**Diabetes**									
No	145 (55.8)	1							
Yes	115 (44.2)	2.529	1.240	5.159	.011	2.669	1.187	6.002	.018
**Hypertension**									
No	134 (51.5)	1							
Yes	126 (48.5)	2.408	1.213	4.779	.012	2.412	1.058	5.496	.036
**Thyroid disorders**									
No	200 (76.9)	1							
Yes	60 (23.1)	0.364	0.184	0.722	0.004	.771	.324	1.837	.557

## Discussion

Currently, the distressingly spread of COVID-19 posed to be a public health emergency across the world. At the moment of writing, no treatment or vaccine that could treat it was discovered. Therefore, prevention is the best solution. Successful prevention and control of COVID-19 are achieved through increasing the population knowledge (especially high-risk groups), attitude, and practice towards COVID-19. To the best of our knowledge, our study is one of the first to examine the knowledge, attitude, and practice variables among individuals with pre-existing comorbidities and their associations with other parameters related to COVID-19. Our study found a high prevalence (82.7%) of adequate knowledge among chronic disease patients. This ﬁnding is significantly higher than the results of studies from Ethiopia, Kenya, China, and Iran, where the authors reported a low prevalence of poor knowledge [9–12]. A possible reason for this could be the difference in the socioeconomic status of study respondents. Moreover, it may also be due to the differences in the tools used for calculations. The studies performed in Iran and China were analyzed during the initial phase of the outbreak when most populations had little or no information about COVID-19.

Akwa *et al*. reported in their study that >80% of respondents knew the disease is spread from person to person and by contact with infectious droplets; also, they were aware of the common symptoms [13]. Our findings were in agreement with theirs since 91.9% of our respondents were well informed. Among all the participants, hypertensive individuals showed better knowledge towards COVID-19 (43.1%) even though a positive attitude was seen among all the comorbidities conditions. In our study, women (48.1%) had an overall better knowledge about COVID 19 compared to men (34.6%). Women depicted a predominantly negative attitude towards the COVID-19 pandemic scenario (60.9%). This could be because, by nature, women tend to socialize a lot more than men. The social isolation and national lockdown strategies by the government prove to be a hindrance for it. Moreover, 49.60% of our respondents felt that the national lockdown and social isolation had worsened their existing comorbidities. Most of our respondents have experienced increased anxiety and depression (23.8% and 10.40%), which is similar to another study [14]. However, the levels of anxiety and depression are much lower than those reported by Roy *et al*., who reported that more than 80% of their participants experienced anxiety and preoccupation with contracting COVID-19 [15].

During this pandemic, most of our participants had difficulty in maintaining a positive attitude towards life (24.60%), followed by those who are facing a financial crisis (20.40%). Also, 19.20% of them faced social isolation, and 19.60% faced difficulty managing household chores. A possible reason for this could be because most of the respondents in our group were either working from home or were above 50 years of age with house help which was now not the norm. In contrast, many individuals (27.7%) were keeping themselves happy by engaging in hobbies and other activities for which they previously had no time.

We also asked our respondents various questions related to the management of their comorbidities. Individuals with thyroid disorders faced significant difficulties in monitoring their condition (p-value=0.001) and had problems accessing a laboratory for frequent testing of thyroid-stimulating hormone (TSH), thyroxine (T4), and triiodothyronine (T3)levels (p-value=0.021). Other comorbidities like diabetes and hypertension were better managed. This can be attributed to increased use of telemedicine and self-monitoring of blood sugar levels. Although the use of smartphone-based consultations has been extensively used during this public health emergency, poor resources like technical glitches, poor quality of audiovisual resources in many parts of India have limited the adequate and satisfactory teleconsultations regarding diabetes education, proper counseling and monitoring of blood pressure.

## Conclusion

All available literature and our study hint that individuals with comorbidities face a higher risk of contracting COVID-19. It is well established that the prevalence of diabetes is highest in Indian COVID-19 patients compared to other countries. However, there is a considerable lacuna of knowledge in the published literature on the prevalence of COVID-19 among individuals with comorbidities from India, even though India ranked second worldwide in that aspect. Considering that India has entered the phase of community spread, patients with COVID-19 could be asymptomatic, like many studies from China, Italy, and Kuwait reported. Hence, countries must adopt a strict policy, especially for the high-risk group, for making testing affordable, imposing home quarantine for asymptomatic cases, and above all, social isolation and wearing face masks outdoors.

## Acknowledgments

### Ethical approval

The approval for this study was obtained from the Ethics Committee of the Amity University, Noida, India (approval ID: AUUP/IEC/2020-September/04AUUP/IEC/2020-September/04).

### Consent to participate

Informed consent was obtained from the participants and their confidentiality was kept.

### Conflict of interest

The authors declare that there is no conflict of interest.
